# Comparison of home range size, habitat use and the influence of resource variations between two species of greater gliders (*Petauroides minor* and *Petauroides volans*)

**DOI:** 10.1371/journal.pone.0286813

**Published:** 2023-10-19

**Authors:** Denise McGregor, Eric Nordberg, Hwan-Jin Yoon, Kara Youngentob, Lin Schwarzkopf, Andrew Krockenberger

**Affiliations:** 1 College of Science and Engineering, James Cook University, Cairns, Queensland, Australia; 2 School of Environmental and Rural Science, University of New England, Armidale, New South Wales, Australia; 3 Health Intelligence, The Australian e-Health Research Centre, CSIRO Health & Biosecurity, Canberra, Australian Capital Territory, Australia; 4 Fenner School of Environment and Society, Australian National University, Canberra, Australian Capital Territory, Australia; 5 College of Science and Engineering, James Cook University, Townsville, Queensland, Australia; 6 Division of Research and Innovation, James Cook University, Cairns, Queensland, Australia; Bowling Green State University, UNITED STATES

## Abstract

Knowledge of the spatial requirements of a species is fundamental to understanding its environmental requirements. However, this can be challenging as the size of a species’ home range can be influenced by ecological factors such as diet and size-dependent metabolic demands, as well as factors related to the quality of their habitat such as the density and distribution of resources needed for food and shelter. Until recently, the genus *Petauroides* was thought to include only a single species with a widespread distribution across eastern Australia. However, a recent study has provided genetic and morphological evidence supporting *Petauroides minor* as a distinct northern species. Previous studies have focused on the ecology of *P*. *volans*, but there has been inadequate research on *P*. *minor*. Data on home range and habitat use were obtained for both species using a combination of techniques including GPS collar locations, radiotelemetry, and spotlighting and comparisons were made using consistent methodology. Home range sizes of *P*. *minor* (4.79 ha ± 0.97 s.d., KUD .95) were significantly larger than those of *P*. *volans* (2.0 ha ± 0.42 s.d., KUD .95). There were no significant differences between male and female home range sizes in either species. Both species showed site-specific preferences for tree species and for larger diameter trees for both forage and shelter. Tree size and biomass/ha were significantly greater in the *P*. *volans* study sites than the *P*. *minor* study sites and there was a negative correlation between home range size and eucalypt biomass. Larger home range size is likely driven by the substantial differences in biomass between northern (tropical) and southern (temperate) eucalypt-dominated habitats affecting the quality and quantity of resources for food and shelter. Understanding landscape use and habitat requirements within each species of *Petauroides* can provide important information regarding limiting factors and in directing conservation and management planning.

## Introduction

Knowledge of the spatial requirements of a species is fundamental to understanding its environmental requirements [1]. Home range is the physical area that an animal uses for the totality of its ecological requirements- food, shelter, and reproduction [2]. The size of a species’ home range is influenced by ecological factors such as diet and size-dependent metabolic demands, as well as the density and distribution of their resources, such as food and shelter [3–5]. Estimates of home range size can be used to help predict population size, carrying capacity [6] and to increase understanding of how the distribution and abundance of required habitat elements, such as tree hollows, influence landscape use. Detailed knowledge of a vulnerable species’ home range and essential resources across its distribution is vital for determining future management choices regarding habitat retention and restoration efforts after anthropogenic disturbance. This is critically important in Australian forests, where recent severe bushfires and ongoing deforestation pose substantial threats to many species, including greater gliders [7].

Until recently, greater gliders were considered a single species, *Petauroides volans*, with a widespread distribution that stretched across eastern Australia from the Windsor Tablelands in north Queensland (Qld), south through eastern New South Wales (NSW), the Australian Capital Territory (ACT), and continuing throughout eastern Victoria (Vic) to Wombat State Forest [8]. However, McGregor *et al*. [9] recently provided genetic and morphological evidence supporting three distinct taxa. *Petauroides minor*, historically considered a subspecies of *P*. *volans* [10, 11], occurs in the north of their range, *P*. *armillatus* is found in central QLD and *P*. *volans* in the south of the historic range. Because the evidence supporting three taxa is new, there are no currently accepted common names for those species and/or subspecies. Consequently, in this paper we refer to all of them collectively as greater gliders.

Previous studies of greater glider habitat use, and home range sizes have been primarily focused on southern populations of *P*. *volans* [12–17] with only a single study on the newly described northern species, *P*. *minor* [18]. The genus *Petauroides* has a widespread geographic range including tropical, subtropical, and temperate regions. This geographic variation, combined with greater gliders’ patchy distribution [19], necessitates a detailed knowledge of habitat requirements for the different greater glider taxa.

*Petauroides* are strictly arboreal and unique among Australia’s gliding mammals for their highly specialized, folivorous diet, dependent almost exclusively on eucalypt leaves for both nutrients and water [14, 20]. In addition, they are dependent on tree hollows for shelter, with individuals reported to use 2–18 hollows [14, 17]. Greater gliders require large hollows with an entrance of >8–10 cm [14, 21]. The formation of these large hollows has been estimated to occur only in eucalypt trees between 220–360 years of age [21, 22]. While greater gliders inhabit a wide range of coastal to montane eucalypt forest and woodland ecosystems, their distribution is often concentrated in small areas of forest that may correspond to higher soil fertility [19] and a diversity of large, mature eucalypts of species providing high levels of foliar nutrients [23]. Forests that support greater gliders are often also highly valued for timber harvesting and agriculture, creating direct competition between land-uses for resources [24–26]. Populations are exceedingly slow to recover from disturbances due to a combination of life history traits including high site fidelity, low reproductive rates, and low dispersal rates [12, 27–30].

Greater glider population declines have been attributed to the cumulative effects of land clearing and the impacts of climate change, including lower than average rainfall and increased occurrence of intense fires [31–35]. An estimated 215,522 ha reduction in greater glider habitat occurred from 2000–2017 from land clearing [36]. More recently, the catastrophic wildfires of 2019–2020, burned almost 1/3 of *P*. *volans* remaining habitat [37]. While there are no population estimates of *Petauroides* across their entire range [38], data from multiple long-term monitoring sites have revealed alarming declines and localized population extinctions over the past thirty years [33, 39–41]. In a comprehensive monitoring program in the central highlands of Victoria, the population of *P*. *volans* declined by 87% over a 22-year period [42]. Similarly, in Booderee National Park, ACT, *P*. *volans* went from being the second most recorded arboreal marsupial to locally extinct within the period from 2004–2007 [33]. In the Lower Blue Mountains, NSW *P*. *volans* have declined at lower elevations in the last 20 years and were no longer detected at 35% of the study sites where they were previously recorded [41]. Similarly, in central Queensland, P. *minor* declines in abundance of 89%, between 1973–76 and 2001–02 were recorded [43]. *Petauroides volans* is currently listed as endangered and *P*. *minor* is listed as vulnerable under the National Environmental Protection and Biodiversity Act [38] and vulnerable globally under the IUCN’s Red List of threatened species [44].

Previous studies provide an incomplete examination of habitat use and home range size across the newly described taxa in the genus *Petauroides* [9]. A greater understanding of how habitat use, and home range differ between *P*. *volans*, and newly designated species of *P*. *minor* will be vital in future management efforts; differentiating high quality habitat in need of protection, from less suitable habitat, and in predicting how each species may respond to future reductions in resources. This is the first study to compare (1) home range size and population density, (2) habitat use preferences for tree species and size, and (3) test for relationships between home range size and resources within and between populations of *P*. *volans* and *P*. *minor* within a single study using consistent methodology.

## Methods

### Study areas

**Fig 1:**
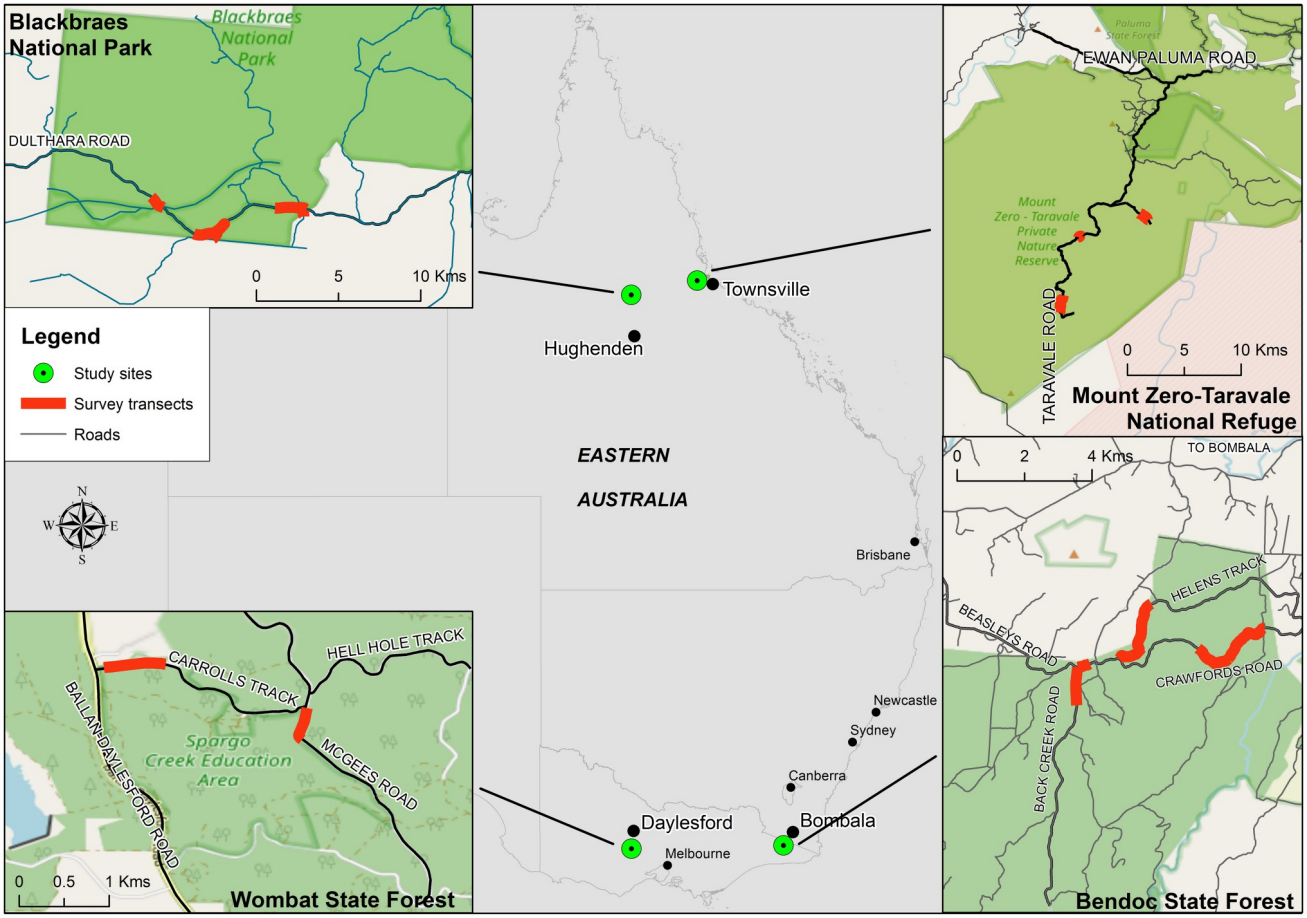
Location of the four study areas in relation to eastern Australia.

This study was conducted at four different locations that broadly represent the extremes of the genus *Petauroides* latitudinal and longitudinal geographic range, with *P*. *minor* found at the two north Queensland sites and *P*. *volans* found at the two Victorian sites (Fig 1). As the sites for this study match collection locations from McGregor *et* al. [9], the species in this study can be unambiguously assigned to *P*. *volans* and *P*. *minor*. Transects were selected at each site as part of a larger study and selection was based on the highest density of greater gliders, as determined during preliminary spotlighting searches to facilitate captures.

The first two sites were in Queensland with populations of *P*. *minor* occupying these locations. Site 1: *P*. *minor* Eastern Site is located in the Mount Zero-Taravale Sanctuary (19°07’18” S, 146°04’42” E), owned and managed by the Australian Wildlife Conservancy, in the Paluma Range, within the Einasleigh Uplands bioregion. Three transects were selected for our study within the 59,000-ha reserve, occurring at an elevation of 518–724 m. Site 2: *P*. *minor* Western Site is located in Blackbraes National Park (19°34′39” S, 144°05′05” E), approximately 200 km west of the Taravale site and 170 km north of Hughenden. The elevation is 1040–1065 m, providing a more variable climate than Site 1.The study area was in the west end of the 52,195 km park, in the Gulf Pains bioregion, along Dulthara Road.

The last two sites were in Victoria and occupied by populations of *P*. *volans*. Site 3: *P*. *volans* Eastern Site is located in Bendoc State Forest (37°10’35” S, 148°56’52” E) and is 85 km west of Eden and 7 km south of the New South Wales border in the Highlands Far East bioregion, elevation 810–930 m. Transects were located along Back Creek Road, Crawford Road and Helen’s Track. Site 4: *P*. *volans* Western Site is in Wombat State Forest (37°29’50” S, 144°09’23” E), 50 km west of Melbourne and 400 km west of Bendoc State Forest. Wombat State Forest is in the Central Victorian Uplands bioregion and straddles the Great Dividing Range. Although large areas of the park were searched for *P*. *volans*, very few individuals were spotted, except for the Spargo Creek Education area (270 h) established in 1987. The study area is located in the south-west corner of the 70,000-ha forest along the Werribee River, elevation 618–639 m. Two transects were selected in this area along McGee track and Carroll track. Additional information on climate, geology and vegetation for each site can be found in S1 Table.

### Spotlighting surveys and density estimates

We conducted spotlighting searches for greater gliders to estimate their densities, record feed trees and to capture them for collaring. Searches occurred between the hours of 20:00 and 02:00 during the following periods: *P*. *minor* Eastern Site (Taravale) 28-10-2015 to 11-12-2015, *P*. *minor* Western Site (Blackbraes) 16-10-2016 to 5-11-2016, *P*. *volans* Eastern Site (Bendoc) 22-03-2016 to 11-05-2016, *P*. *volans* Western Site (Wombat) 17-05-2016 to 15-06-2016. Transects were traversed with a slow-moving vehicle (5 mph) using high-powered, handheld torches (Ledlenser P7) to detect greater glider eye shine. To reduce the potential for unintended bias, we searched each transect on three nights, with order and direction varied each night. Two searchers worked as a team spotlighting opposite sides of the track. When a greater glider was sighted, the location was recorded with a hand-held GPS (Global positioning system; Garmin Oregon 650, Garmin International Inc., KS, USA). The perpendicular distance from the transect line to each animal’s location was measured with a digital laser (Rangefinder 300 XL Opti-Logic).

Line transect sampling was chosen to estimate density because it allows for all detected animals to be recorded, making it more efficient than quadrant sampling for sparsely distributed animals, providing a larger sample size for the same search effort (Buckland 2001). The density of greater gliders was estimated, using the conventional Distance Sampling engine with Distance version 7.1 software [45], for multiple transects at each study site along tracks where gliders were frequently seen in previous searches (transects: Taravale-3, Blackbraes 4, Bendoc 3, Wombat 2). The average density across all transects within each of the four sites was also calculated. Greater gliders observed on both sides of the transect line, with a line length *L*, were counted to a distance of *w*, with *w* being the maximum distance greater gliders were detected from the line. The sampled area *(a)* was defined as *a =* 2*wl*. Density was estimated according to the general equation D = *n* /(*2wLP*_*a*_*)* where *n* number of objects at selected distances *x*_*1*_, x_2_,…x_n_ and *P*_*a*_ is the proportion of objects we expect to detect in this area. The two critical assumptions required to ensure a reliable estimation of density from line transect sampling are: all animals directly on the transect line are always detected (probability = 1) and animals are detected in their initial locations prior to any movement in response to the observer [46]. Greater gliders have characteristic bright white or yellow eye shine, making them one of the most readily detected species during spotlight searches [47]. They are also large and slow moving, when not gliding, and did not flee during spotlight searches. Therefore, repeat counts of the same individual on a given transect, or failing to detect individuals on the transect line was unlikely to occur. A detection function was determined using Distance 7.1, with data pooled from all transects at each study site, to provide an increased sample size for greater accuracy. Three detection function models (half-normal, uniform and hazard rate) for the detection function were fitted to the data using three adjustable expansions sequentially (cosine, simple-polynomial, and hermite-polynomial) with the choice of model based on the lowest value of Akaike’s information criterion (AIC) and the fit of models estimated using chi-square goodness of fit tests [46]. To improve the fit of the model, greater gliders measured at the extreme edges of the area were considered outliers and eliminated. These included less than 5% of the observations [46]. Greater glider densities from these searches should not be extrapolated over larger areas, as their distribution is known to be patchy and these areas were selected specifically for their high densities, as part of a larger study.

### Animal capture, collaring and radio tracking

Greater gliders are difficult to capture given that their movements are confined to the forest canopy and as obligate folivores they are difficult to lure into traps with food [12, 30]. In this study, gliders were located using the previously described spotlighting, and were captured using a gas-powered, tranquilizer dart-gun (Montech Black Wolf; Tranquil Arms Company, VIC, Australia) as described in McGregor *et al*. [9]. Capture was attempted only when the greater glider was in a branch from which it could be safely caught and positioned facing away from the shooter with only its posterior exposed. In a previous study, using the same sedative on another Pseudocheirid species (*P*. *archeri*), animals were fully alert and active 2 hours after sedation [48]. Although there have been no studies of the pharmacokinetic properties of these drugs with marsupials, a review of pharmacology and use across a wide range of mammals reported plasma half-lives of 1 to 5 hours [49].

While still under sedation, captured individuals were weighed, sexed and external measurements were taken as described in McGregor *et al*. [9]. *Petauroides volans* larger than 1000 g body mass were classified as adults, and smaller individuals were classified as juveniles [50]. We considered *Petauroides minor* adults if individuals were 550 g or more, because only females over 550 g had offspring in this study. After measurements were completed, animals were placed in a hessian bag and transported (<15 km) to a nearby field station where they were held for a day, as part of separate study of metabolic rate. Captured animals were fitted with GPS telemetry collars, with VHF beacons (Sirtrack LiteTrack 30), weighing 28g (<5% of body weight) and released after dark onto the same tree from which they were taken. All animals were released within 24 hours of capture.

A total of 34 greater gliders (12 males and 22 females) from 4 sites *P*. *minor* Western Site (Taravale) N = 12, *P*. *minor* Eastern Site (Blackbraes) N = 11, *P*. *volans* Western Site (Bendoc) N = 5, *P*. *volans* Eastern Site (Wombat N = 6, were fitted with collars. At the Taravale site, collars were equipped with external whip antennae. However, these antennae broke in 8 of 10 collars deployed and were replaced with internal antennae before use at the remaining three sites. Collars were programmed to record nighttime locations every hour from 19:00 to 06:00 for 6 weeks after release. A longer period of tracking would have been preferred but weight restrictions for the collars limited the battery size and therefore the period of continuous tracking. Early trials showed that GPS collars were unable to connect with satellites when greater gliders were in their dens. However, the collars’ VHF beacons were active daily between 10:00 to 14:00 during the same six-week period. This allowed greater gliders to be tracked during the day to their dens with a hand-held radio receiver (H.A.B.I.T Research Ltd., Canada, model HR2500) and 3-element Yagi antennae. After this period, the VHF beacon was active from 18:00 to 02:00 to assist in tracking greater gliders for recapture to allow for collar removal.

### Home range size calculations

Home range size estimates for greater gliders were calculated using GPS collar location data. Comport *et al*. [18] found 1-hour intervals were sufficient to provide independence of consecutive locations for greater gliders. To confirm the suitable period between locations for this study, preliminary analysis was conducted on a subset of greater gliders (8 gliders, 2 per study area). The percentages of consecutive relocations occurring at 1-hour intervals in which the individual moved more than 6 meters were calculated (6 m was adopted because tree canopies were generally less than 6 m and GPS precision was generally around 6m). On average, 90% (ranging from 83% to 92%) of hourly sequential locations were at least 6 m apart, suggesting that an hour was sufficient for gliders to move between trees. Given that single-glide distances by greater gliders can be a substantial proportion of the home range diameter, hourly locations were sufficiently independent for calculating home range size in this study.

The location data was screened for errors by eliminating all two-dimensional locations (3 satellites) and three-dimensional data (4 or more satellites) with a PDOP greater than 10 [51]. Trees used for daytime shelter by nocturnal species are an essential component of their home range [52, 53]. Therefore, diurnal den locations, obtained from radiotelemetry, were included, once per den, in the location data. We conducted home range size analyses using the ZoaTrack platform [54]. We used multiple methods to estimate home range area, to allow for broader comparability with previous *Petauroides* studies [12–15, 17, 18, 28]. We included fixed-kernel utilization distribution (KUD) estimates calculated at 95%, 50% isopleths and minimum convex polygon estimates (MCP) [55] at 95% and 100% isopleths. For KUD a constant smoothing factor *h*_*ad hoc*,_ (h = 20) and a grid = 300 was used for all home range size calculations. This allowed for a more accurate comparison across individuals within and between locations in our study, as bias associated with the smoothing factor was uniform. We derived the smoothing factor by the visual comparison of all home range size estimates at differing smoothing factors in increments of five percent. The smoothing factor chosen minimized the inclusion of excessive areas around each sample point while avoiding fragmentation in home range size estimates centred on the most frequented locations [56, 57].

The influence of sample size on home range size estimates was assessed using asymptote analysis on GPS telemetry location data [58, 59]. For each glider, a routine was created in R using the adehabitatHR package [60], that randomly selected locations from the full data set, with replacement, in increments of ten (20, 30, 40 etc up to all locations). For each possible sample size, an estimate of home range size was calculated (95% KUD) with 10 iterations and the means of the cumulative home range areas were plotted against sample size to produce an asymptote curve with standard errors.

The average minimum nightly distance travelled was calculated for each greater glider where a minimum of five locations per night were recorded for 5 or more nights. Minimum distance travelled was calculated by the sum of the distances between each sequential recorded location. We were unable to determine the maximum distance travelled per night or the maximum distance per glide, because our data does not provide information on their movements between GPS locations.

#### Statistical analysis

We used linear models with home range size (at KUD 95%, KUD 50% and MCP 95%) each as the response variable and species and sex and the interaction between species and sex as the explanatory variable. Post-hoc comparisons between *P*. *volans* and *P*. *minor* were done at the KUD 95%, and 50% isopleths and MCP at the 95% isopleth. R software was used for these analysis [60].

### Measurements of resource availability and tree preference for food and shelter

We estimated resource availability from a census of tree stand composition within 48 plots (60m X 60m, 12 per study site). Tree species, height, and diameter at breast height (DBH; measured 1.3 m from the forest floor on the uphill side of the tree) were recorded for all trees within the plot with a minimum diameter of 7 cm, using a digital laser (Rangefinder 300 XL Opti-Logic) and DBH measuring tape, respectively. Plots were positioned to overlap greater glider home ranges. The location of each plot was chosen by randomly selecting 48 centre points from all GPS locations where *Petauroides* were observed foraging during spotlight searches.

Greater glider selection of food trees was determined by direct observation of browsing animals during repeated spotlight searches. These trees were marked, given an identification number and their locations recorded using a hand-held GPS (Garmin Oregon 650, Garmin International Inc., KS, USA). Browse trees were revisited during the day, recording tree species, height and DBH. Leaf samples were collected from browse trees for a separate study. To determine greater gliders’ preferences to forage in specific tree species or tree size classes, the proportion of feeding selections in each tree species was compared to availability of each species in the sample plots at each site [18]. Similarly, we investigated the relationship between the DBH of trees in which greater gliders were observed to feed, compared to what was available in the plots using the same methodology. Trees were grouped by DBH into one of the following five categories: 7–30 cm, 30-50- cm, 50–70 cm, 70–90 cm, greater than 90 cm. Trees with a DBH of less than 7 cm were not recorded.

Trees selected by greater gliders for dens were located by daytime tracking of collared animals and given an identification number and their location was recorded with a hand-held GPS (Garmin Oregon 650, Garmin International Inc., KS, USA). Two observers watched the hollow simultaneously with binoculars, commencing an hour before dusk, until the collared animal emerged, to confirm dens. Tree species, height, DBH, den height and den aspect were recorded for all trees with confirmed dens. To determine greater glider preferences to shelter in specific tree species and tree size classes, the proportion of dens in each tree species and the DBH class of each den tree was compared to the availability of each tree species and DBH class in sample plots.

#### Statistical analysis

We used Chi square goodness of fit tests to make comparisons at each site, between the proportions of each tree species and tree diameter classes observed being used by greater gliders for feeding and for dens to the expected proportions of each tree species and tree diameter class available in sample plots. Expected proportions of trees of each tree species and diameter class were estimated based on our plot surveys as described above. S-Plus 8.0 software was used for the analyses.

### The relationship between home range size and environmental variables

The environmental variables were estimated from the survey plots described above.

Aboveground tree biomass (AGB) was estimated for each plot using the tree-based allometric equation:

$$\text{ln}\left(\text{AGB}\right)=-2.0596+2.1561\;\text{ln}\left(\text{D}\right)+0.1362{\left(\text{ln}\left(\text{H}\right)\right)}^{2}$$

[61]

ABG was measured in kg tree^-1^, with D and H the measurements of tree DBH and height measured in centimetres and meters, respectively. Only trees with a DBH of 7 cm or more were included in this analysis. This model was highly reliable across species and sites when used to estimate AGB of eucalypt woodlands in eastern Australia [61]. The measure of ‘total biomass’ was estimated by summing the biomass of all trees in sample plots. The ‘biomass of tree species preferred for foraging’ and the ‘biomass of tree species preferred for dens’ were estimated by extracting data from the ‘total biomass’, based on greater glider preferences for tree species and DBH documented at each site through feeding and denning observations. Data collected from feeding observations was used to determine greater glider preferences for tree species and the minimum diameter (per species) in which they were known to feed at each site. All trees from sample plot surveys, meeting this selection category, were then included in totals of biomass of tree species preferred for foraging. Similarly, to estimate the biomass of tree species preferred for dens, greater glider’s preferences for tree species and minimum dbh (per species) was derived from trees observed to contain active greater glider dens at each site.

Annual precipitation for each animals’ home range location, was estimated by extracting point data using R version 3.4.0 and packages ’sp’ (function SpatialPoints()) and ’raster’ (function extract()) from climate layers created using ANUCLIM 6.1 software [62] based on a 250m resolution (0.0025 decimal degrees) digital elevation model from Geoscience Australia [63].

#### Statistical analysis

We used Akaike’s Information Criterion (AIC) to pick the best model with individuals’ home range size (95% KUD) as a response variable and environmental variables as explanatory variables that might, a priori, be associated with greater glider habitat quality. The explanatory variables included were total biomass, biomass of trees species preferred for foraging, biomass of tree species preferred for dens, animal density and annual precipitation. Of the 31 models evaluated, the best model was total biomass (AICc = -1.252, S2 Table). We then used a GLM to assess the relationship between home range size (95% KUD) and total biomass. R software was used for this analysis [60].

### Permits and ethical statement

Research was conducted under permits from Victoria Department of Environment, Land, Water and Planning (permit # 10007842), and Queensland Scientific Purposes Permits (permit #s WITK16408715, WISP16408815, WIF416492015, WITK18792718, WISP18803718, WIF418792618). This study complied with the legal and ethical requirements of the state of Queensland and was conducted under approvals from the JCU Animal Ethics Committee (permit # A2137).

## Results

### GPS location data

Thirty-four collars were fitted to greater gliders, however, 15 had less than optimal performance (8 collars fell off ≤ 5 days after animals were released, 3 had insufficient numbers of locations, 3 could not be located with VHF and 1 animal was consumed by a python). Home range size estimates were calculated for the remaining 19 animals (9 *P*. *minor*, 10 *P*. *volans*, Table 1). The number of GPS telemetry locations per animal ranged from 37–512 per collar. Individuals of *P*. *minor* were tracked nightly for a mean of 34 days and 323 locations and individuals of *P*. *volans* were tracked nightly for a mean of 37 days and 223 locations [55, 58, 59]. We conducted asymptote analysis for every glider to establish the minimum sample size needed to estimate home range size. The minimum number of locations to reach an asymptote varied between individuals, occurring between 35–100 independent locations using 95% KUD method (S1 Fig). The 19 animals included in our analysis showed a clear asymptote in home range size prior to reaching the maximum number of GPS telemetry locations in each respective data set. Due to a programming error, the collar on animal WG1 recorded one location daily instead of hourly. This individual was included with only 37 locations because there was a clear asymptote at 30 locations.

**Table 1:** Home range size and core estimates for nine *P*. *volans* and ten *P*. *minor* calculated using the fixed kernel method at 95% isopleth and the minimum convex polygon method.

Species	Location & Glider ID	Mass (g)	Sex	KUD 95%	KUD 50%	MCP 95%	MCP 100%	GPS fixes	Days tracked	Min. nightly distance ± s.e.
***P*. *minor***	**Blackbraes, Qld**	**665**						**181**		
	BG8	685	M	4.2	0.8	2.5	4.2	88	10/27/16-11/12/16	1143 ± (345)
	BG3	645	M	5.8	1.1	4.7	13.4	274	10/20/16-11/17/16	879 ± (184)
	BG14 *	490	JM	1.3	0.3	0.3	0.6	66	11/04/16-11/14/16	530 ± (108
***P*. *minor***	**Taravale, Qld**	**712**						**371**		
	TG12	695	F	6.5	1.0	4.3	12.8	297	12/04/15-01/13/16	707 ± (92)
	TG6	730	F	3.7	0.8	2.1	12.9	412	11/22/15-01/06/16	751 ± (73)
	TG5	777	F	4.8	0.8	3.0	21.5	427	12/02/15-01/13/16	512 ± (69)
	TG11	660	M	5.1	1.1	4.0	9.8	456	12/02/15-01/13/16	514 ± (53)
	TG8	740	M	4.2	0.6	2.6	14.2	512	11/26/15-01/10/16	672 ± (82)
	TG4	670	M	4.0	0.4	2.0	7.3	120	11/18/15-12/11/15	723 ± (176)
***P*. *volans***	**Bendoc, Vic**	**1444**						**250**		
	EG4	1445	F	2.0	0.4	1.0	1.5	415	04/06/16-05/25/16	215 ± (25)
	EG5	1280	F	1.7	0.4	0.4	0.5	38	04/10/16-04/16/16	133 ± (60)
	EG7	1565	F	1.5	0.3	0.7	1.9	243	05/09/16-06/12/16	411 ± (73)
	EG8	1485	M	1.6	0.3	0.7	1.3	305	05/12/16-06/18/16	448 ± (73)
***P*. *volans***	**Wombat, Vic**	**1293**						**205**		
	WG1	1500	F	2.2	0.5	0.7	0.8	37	05/22/16-06/28/16	
	WG2	1340	F	1.5	0.4	0.4	0.7	211	05/24/16-07/04/16	301 ± (44)
	WG3	1330	F	2.3	0.5	1.6	1.7	375	06/04/16-07/16/16	308 ± (25)
	WG5	1330	F	2.1	0.5	1.1	1.3	275	06/10/16-07/23/16	282 ± (29)
	WG4	1015	F	2.6	0.4	1.4	1.9	283	06/06/16-06/16/16	208 ± (27)
	WG6	1240	M	2.6	0.6	1.0	1.2	49	06/14/16-06/20/16	246 ± (28)

Mass refers to glider body mass in grams (g). Abbreviations for sex are M = male, F = female, and JM = juvenile male. KUD is kernel utilization distribution (50%, 95%) estimates in hectares. MCP is minimum convex polygon estimates in hectares (95%). The dates tracked are provided as month/day/year. Bolded numbers in the table show the data means for each location. *BG14 was a juvenile and therefore not included in further analysis of home range size or minimum nightly distance traveled. WG1 was not included in minimum nightly distance calculations because there were not five locations recorded for a minimum of five nights.

### Home range size estimates

Home range size estimates for *P*. *volans* ranged from 1.5 to 2.6 ha (95% KUD) with a mean of 2.0 ha (standard deviation (s.d.) 0.42) and for *P*. *minor* ranged from 3.7 to 6.5 ha (95% KUD) with a mean of 4.79 ha (s.d. 0.97). Estimates using MCP (95%) for *P*. *volans* ranged from 0.4 to 1.6 ha (mean 0.89 ha, s.d. 0.40) and for *P*. *minor* ranged from 2.0 to 4.7 ha (mean of 3.15 ha, s.d. 1.04). The home range sizes of *P*. *minor* were significantly larger than those of *P*. *volans* at all three KUD isopleths (95%, 50%) and when compared using MCP at the 95% (Table 2). We found no differences between males and females home range sizes within species and no interaction between home range size, species, and sex (Table 2). The average minimum nightly distance travelled by *P*. *minor* was 740 m (± 72.08 s.e.), over twice as far as *P*. *volans* with an average minimum nightly distance of 284 m (± 31.15 s.e.; Table 1).

**Table 2:** Results from linear models with home range size (at KUD 95%, KUD 75%, KUD 50% and MCP 95%) each as the response variable and species and sex and the interaction between species and sex as the explanatory variable. Post-hoc comparisons between *P*. *volans* and *P*. *minor* were done at the KUD 95%, and 50% isopleths and MCP at the 95% isopleth.

Variable	KUD 95		KUD 50		MCP 95	
	Estimate ± (se)	p-value	Estimate ± (se)	p-value	Estimate± (se)	p-value
Species	-3.01 (0.51)	<0.001	-0.44 (0.13)	0.004	-2.22 (0.54)	<0.001
Sex	-0.34 (0.55)	0.546	-0.07 (0.14)	0.632	0.027 (0.59)	0.964
Species x Sex	0.453 (0.81)	0.585	0.09 (0.20)	0.655	-0.139 (0.86)	0.875
Post-hoc test (Species)						
Comparison	KUD 95		KUD 50		MCP 95	
	Diff of means ± (se)	p-value	Diff of means ± (se)	p-value	Diff of means ± (se)	p-value
*P*.*m–P*.*v*	2.79 (0.41)	<0.001	0.40 (0.10)	0.001	2.29 (0.43)	<0.001

**Fig 2:**
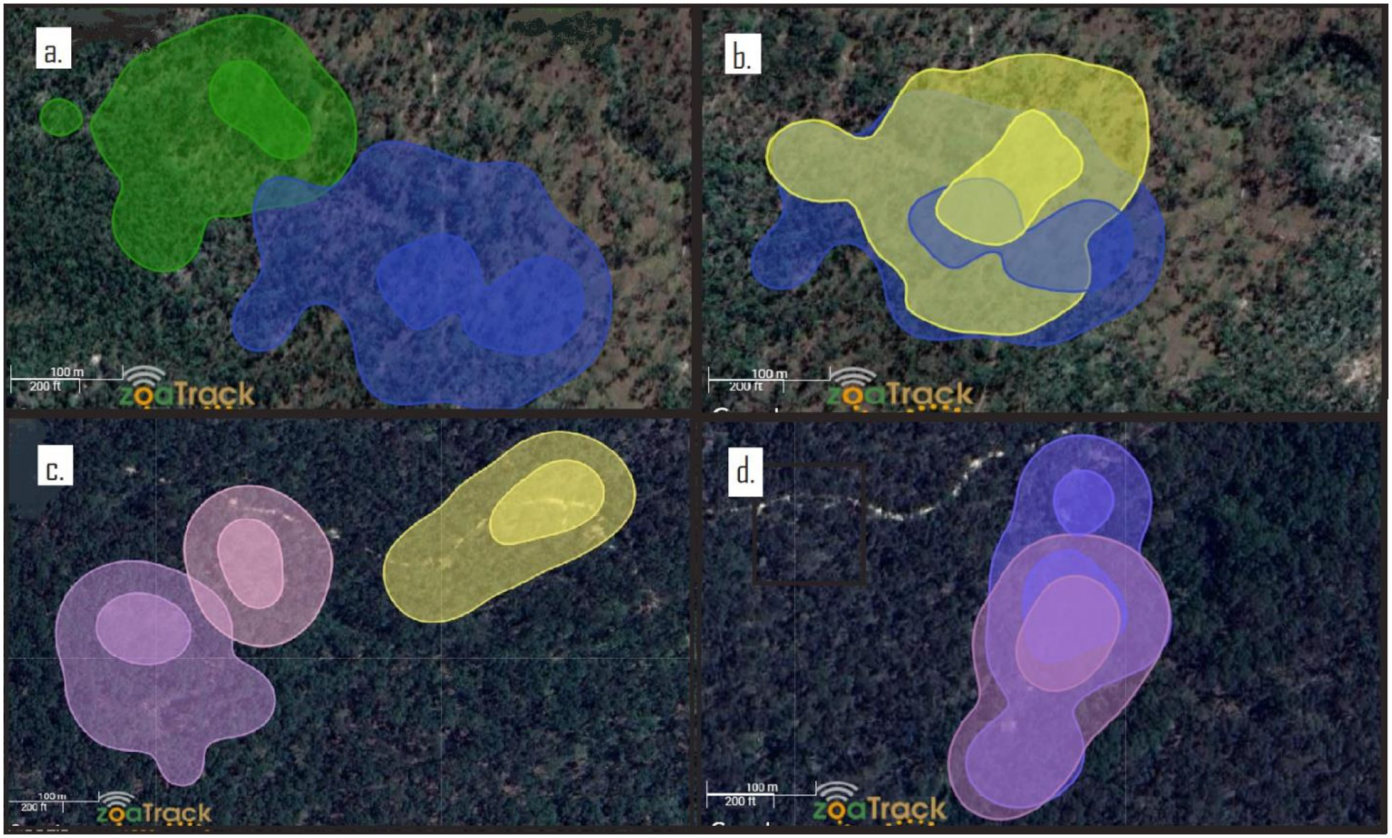
Home range overlap between individual greater gliders. Overlay of KUD home range areas at 95% isopleth (light shades) and core 50% isopleth (bold shades) a.) *P*. *minor*-Eastern Site (Taravale): adult male (TG11, blue) and adult male (TG4, green). b.) *P*. *minor*-Eastern Site (Taravale): adult female (TG5, yellow) and adult male (TG11, blue). c.) *P*. *volans*-Western Site (Wombat): three adult females (WG2, yellow; WG1, purple; WG4, pink). d.) *P*. *volans*-Eastern Site (Wombat): adult female (WG2, purple) and adult male (WG3, blue).

A thorough analysis of home range overlap was not possible, as not all greater gliders in each area were collared. However, in several instances, it was possible to overlay home ranges when animals occupied adjacent areas. This revealed a pattern that was consistent with discrete home ranges between neighbouring males and usually between neighbouring females, and an extensive overlap of home ranges between males and females, including core areas (Fig 2). Spotlighting observations recorded limited occurrences of simultaneous co-use of trees by two or three greater gliders (*P*. *minor*: 7% Taravale, 9% Blackbraes; *P*. *volans*: 11% Bendoc, 13% Wombat).

### Animal density

We sited 325 *Petauroides* in repeated transect surveys to estimate animal density (*P*. *minor*: Western Site (Taravale) n = 69, Eastern Site (Blackbraes) NP n = 147, an *P*. *volans*: Western Site (Wombat) n = 33, Bendoc SF n = 76). Density estimates varied within and between study sites, with the greatest density of 2.49 animals per hectare occurring in the 270 h Spargo Creek Education Area of Wombat State Forest (Table 3). The lowest density of animals occurred at Bendoc, Victoria, where the density was 0.61 animals per hectare.

**Table 3:** Animal density and estimated population abundance for *P*. *minor* at Eastern Site (Taravale) and Western Site (Blackbraes) and *P*. *volans* at Western Site (Bendoc) and Eastern Site (Wombat) by search transect and site.

Species	Study site	Search area (ha)	Density (per ha) (95% CI)		Population est. (95% CI)	
***P*. *minor***	**Taravale AWC**	**27.4**	**0.96**	**(0.77–1.19)**	**26**	**(21–33)**
	Creek Track	2.1	2.59	(2.08–3.23)	6	(4–7)
	Hellhole Track	14.8	0.84	(0.68–1.05)	12	(10–16)
	Return Creek Track	10.5	0.79	(0.63–0.98)	8	(7–10)
***P*. *minor***	**Blackbraes NP**	**41.6**	**1.92**	**(1.52–2.43)**	**96**	**(76–122)**
	Dulthara Road 1	15	2.41	(1.90–3.05)	36	(29–46)
	Dulthara Road 2	5.6	1.39	(1.10–1.76)	8	(6–10)
	Dulthara Road 3	16.6	1.14	(0.90–1.45)	19	(15–24)
	Track 11	4.4	3.40	(2.69–4.31)	15	(12–19)
***P*. *volans***	**Bendoc SF**	**41.7**	**0.61**	**(0.51–0.73)**	**25**	**(21–30)**
	Crawford Track	16	0.93	(0.78–1.10)	15	(12–18)
	Back Creek Road	11	0.32	(0.27–0.38)	4	(3–4)
	Hellen’s Track	14.7	0.48	(0.40–0.57)	7	(6–8)
***P*. *volans***	**Wombat SF**	**4.4**	**2.49**	**(1.89–3.28)**	**11**	**(8–14)**
	McGee Road	1.5	3.48	(2.63–4.59)	5	(4–7)
	Carroll’s Track	2.9	1.98	(1.50–2.61)	6	(4–8)

Abbreviations are confidence interval (CI), and hectare (ha) Estimated population is derived from the search area and the density. Bolded numbers in the table show the data total (Search area and Population est.) and means (Density) for each bolded location (Study site).

### Resource availability and tree preference

**Fig 3:**
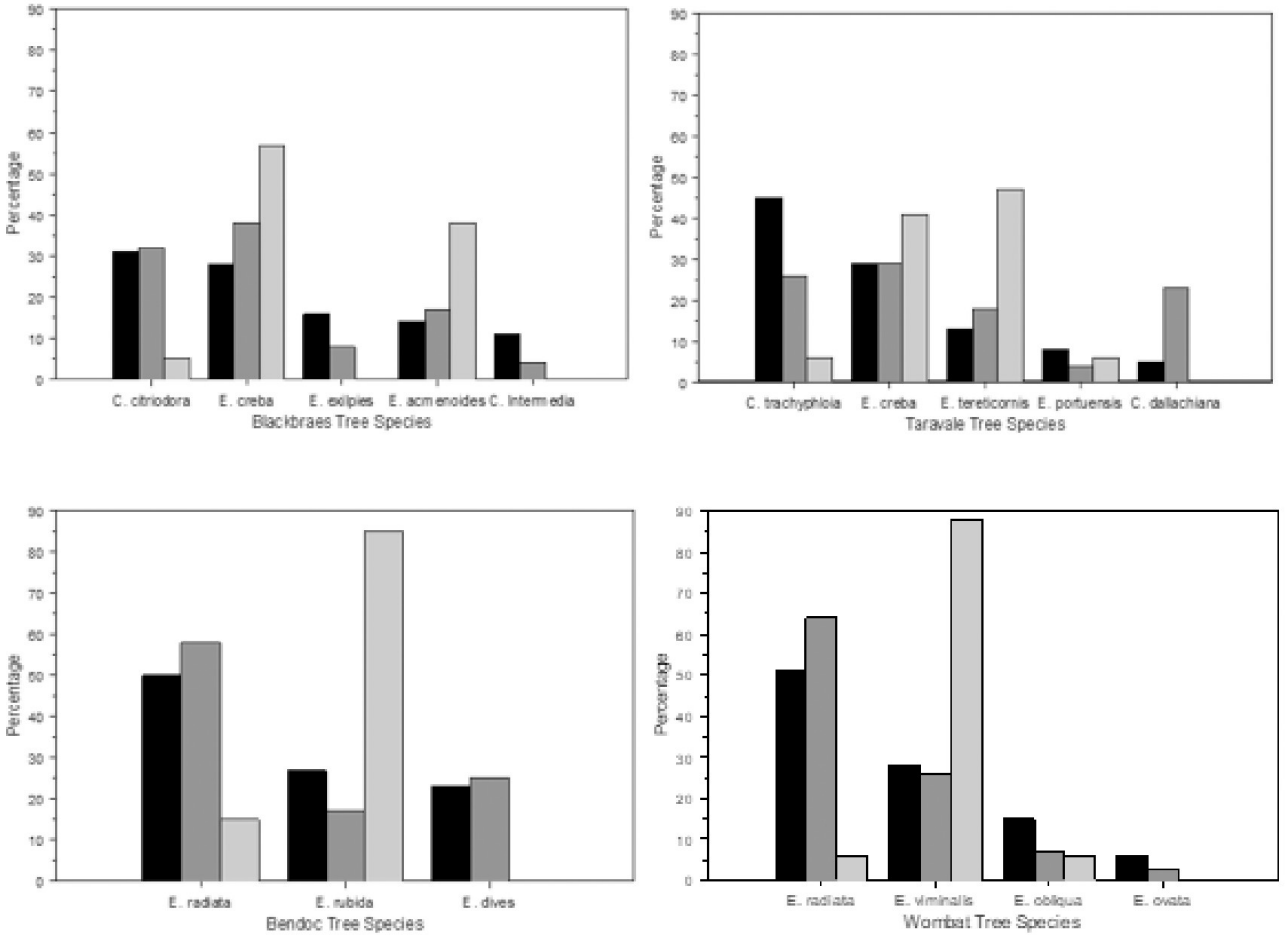
Comparison of tree species available to percentages of species selected for foraging and shelter in each cite: *P*. *minor* Eastern site (Taravale), *P*. *minor* Western Site (Blackbraes) and *P*. *volans* Eastern Site (Bendoc) and *P*. *volans* Western Site (Wombat). Black bars are the percentages of each tree species in survey plots (total n per study area = 466, 432, 1280, 1046, respectively). Dark grey bars are the percentages of each tree species used as feed trees (total n per study area = 102, 94, 46, and 70, respectively). Light grey bars are the percentage of each tree species used for dens (total n per study area = 21, 17, 13, and 16, respectively).

During this study, 312 (*P*. *minor*: Western Site (Taravale) n = 94, Eastern Site (Blackbraes) NP n = 102, an *P*. *volans*: Western Site (Wombat) n = 70, Bendoc SF n = 46) nighttime sightings of greater gliders foraging in trees were recorded. The composition of tree species varied across sites, and greater gliders did not forage in trees in proportion to their abundance in the study areas (Fig 3; *P*. *minor*: Eastern Site (Taravale), χ^2^ = 106.45, df 3, P < .001; Western Site (Blackbraes), χ^2^ = 12.27, df 3, P = 0.007; and *P*. *volans*: Eastern Site (Bendoc), χ^2^ = 6.89, df 1, P = 0.009; Western Site (Wombat), χ^2^ = 7.27, df = 2, P = 0.03). *Petauroides volans* was strongly dependent on *E*. *radiata* at both Bendoc and Wombat accounting for 54% and 55% of foraging observations, respectively, but this was only a slight preference above expected within these sites as this species was also the most commonly occurring species in these survey plots, comprising 50%, and 51%, respectively. *Petauroides minor* at Taravale, showed a strong preference for foraging in *Corymbia dallachiana*, which accounted for 23% of foraging observations but only 5% of the total tree species in survey plots. At Blackbraes, they showed the strongest preference for *Eucalyptus crebra*, with 38% of foraging observations but only 28% of the total tree species in survey plots.

Greater gliders at all sites preferred to forage in trees with larger diameters than would be expected if trees were chosen at random *P*. *minor*: Eastern Site (Taravale) χ^2^ = 32.71, df 2, P = <0.001; Western Site (Blackbraes) χ^2^ = 337.85, df 2, P = <0.001 and *P*. *volans*: Eastern Site (Bendoc) χ^2^ = 112.58, df 2, P = <0.001; Western Site (Wombat) χ^2^ = 185.54, df 3, P = <0.001). *Petauroides volans* were observed feeding in trees with a diameter >70 cm 55% the time, although that size class represented only 12% of the trees in survey plots. *P*. *minor* foraged in trees with a diameter >50 cm more than 50% of the time, although that size class represented only 14% of the trees present. Trees with a diameter of >70 represented only 1% of the total stand at the Queensland sites. The most common diameter of trees at each site were in the 7–30 cm class (50–63%). However, trees of this diameter were relatively avoided by *P*. *volans* where foraging occurred only 40% at the Eastern Site (Taravale) and 16% of foraging at the Western Site (Blackbraes). A higher frequency of foraging in small diameter trees at Taravale may have been due to the strong preference for *C*. *dallachiana*, a medium-sized tree.

#### Tree preferences for shelter

**Fig 4:**
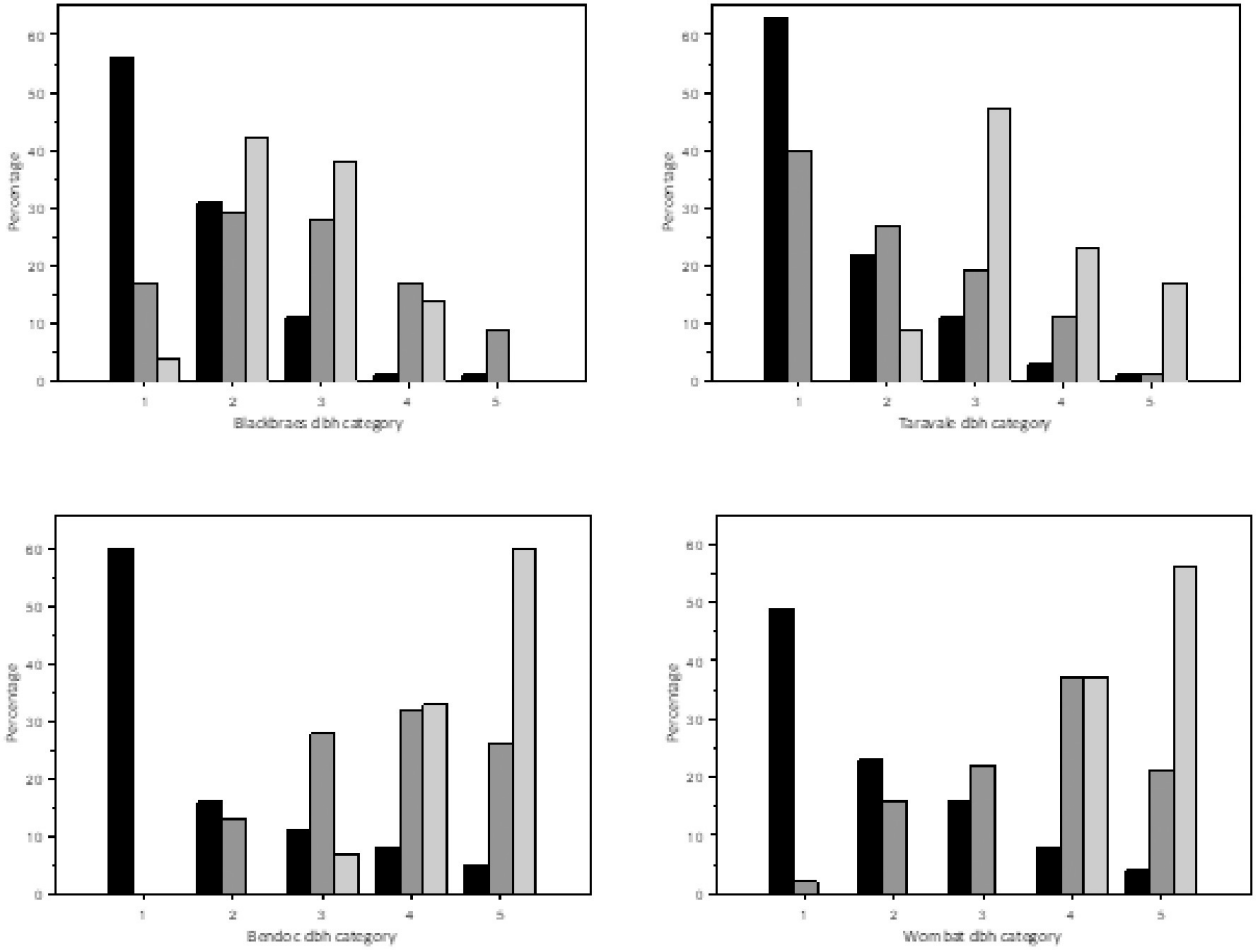
Comparison of the percentage of live trees available in each of 5 DBH categories (7–30 cm, 30-50- cm, 50–70 cm, 70–90 cm, > 90 cm) in survey plots to the percentage of trees of each size used as feed trees and for shelter at each site. Black bars are the percentages of trees available in survey plots in each DBH category (total n per study area = 466, 432, 1280, 1046, respectively). Dark grey bars are the percentage of trees in each DBH category used for feed trees (total n per study area = 102, 94, 46, 70, respectively. Light grey bars are the percentage of trees in each DBH category used for dens (total n per study area = 21, 17, 15, 16, respectively).

In total, 32 greater gliders were tracked to 77 distinct dens (in 76 different trees), across the four study sites: (P. minor; Eastern Site (Taravale) N = 22, Western Site (Blackbraes) N = 22, and P. volans: Eastern Site (Wombat) N = 18, and Western Site (Bendoc) N = 15). Greater gliders occupied 1–6 dens each, over the course of radio tracking. The number of dens used per glider continued to increase with the total number of days tracked. This suggests that more radio tracking days would be required to record the maximum number of dens used per individual. Tree species selection by greater gliders for dens appears to differ from the availability in which tree species are present in the habitats (Fig 4). However, because of low expected frequencies of dens in most tree species, analysis with chi-square goodness of fit test was not possible. *Petauroides minor* used hollows for shelter in *E*. *tereticornis* and *E*. *creba* at the Western Site (Taravale) and *E*. *creba* and *E*. *acmenoides* at the Eastern Site (Blackbraes). *Petauroides volans* predominantly used hollows in a single tree species at each site, *E*. *rubida* at the Eastern Site (Bendoc) and *E*. *viminalis* at The Western Site (Wombat), although each species represented only approximately 25% of trees available in survey plots. Dead trees, many of undetermined species, were also used for dens: 14% Blackbraes, 23% Taravale, 11% Wombat, and 0% Bendoc.

Greater gliders selected trees in the largest diameter classes for dens at a frequency much higher than what would be expected based on their limited availability in survey plots. *Petauroides volans* selected hollows in trees with a diameter >70 cm in 93% of the dens we located, although the availability of this size of trees in survey plots was only 12%. *Petauroides minor* selected hollows in trees with a DBH >50 cm in 70% of dens located, which comprise only 14% of trees available in survey plots. *Petauroides volans* dens were absent from trees with a DBH of <56 cm and *P*. *minor* dens were absent from trees with a DBH <38 cm. We were unable to use a chi square GOF test due to low number of expected frequencies of dens in higher DBH classes. The mean DBH of trees selected by *P*. *volans* for dens in Victoria (93.71 cm ± 4.19 s.e.) was significantly larger than the trees selected by *P*. *minor* (71.40 cm ±3.71 s.e.; *t* = -3.99, P<0.001). Trees selected for dens by *P*. *volans* had a mean height of 31 m (range 20.6–42.1, s.e. 1.24) with the mean height of den entrances at 17.34 m (range 6.9 m—26.8 m, s.e. 1.13). Trees used for dens by *P*. *minor* had a mean height of 23 m (range 14.1–41.1, s.e. 0.84) with entrances at a mean height of 15.15 m (range 7.6 m—24.6 m, s.e. 0.80).

### Relationships between home range size and habitat variables

**Fig 5:**
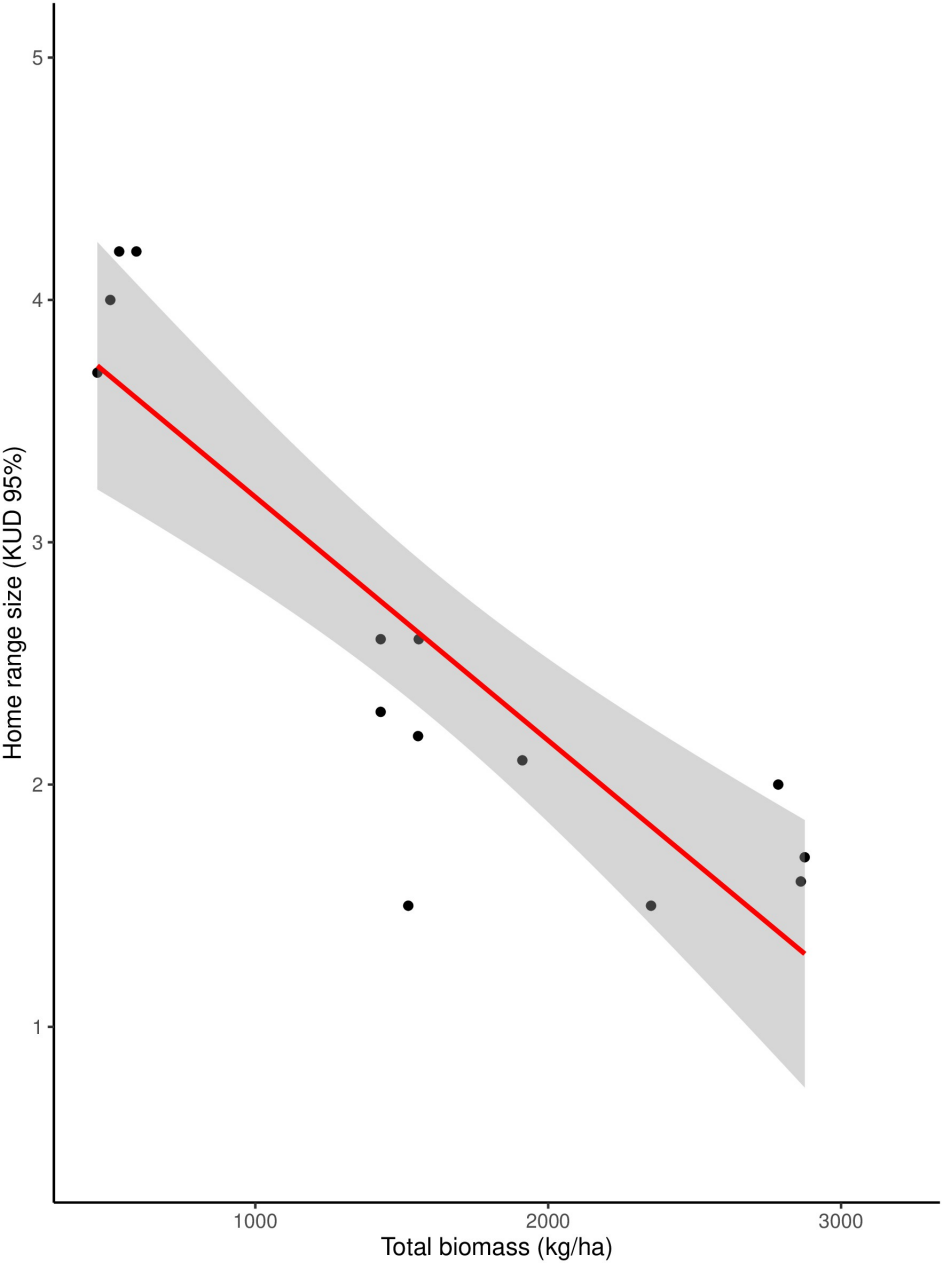
Relationship between *Petauroides* home range size and the den tree biomass of survey plots. Home range size is measured in hectares and biomass is measured in kg/hectare.

We found a significant effect of total biomass on home range size (p-value <0.001). Total biomass accounted for 66% of the total variation in home range size (Fig 5).

## Discussion

### Home range size estimates

This study is the first to directly compare the home range sizes of *P*. *volans* and *P*. *minor*, implementing consistent methodology and analysis techniques. To date, most studies have focused on populations of *P*. *volans*. The effective management and conservation of *P*. *minor* requires species-specific data on their ecology and how they may differ from *P*. *volans*. We found that home range sizes of northern populations of *P*. *minor* were nearly double those of southern populations of *P*. *volans*. Interspecific variations in home range sizes are partly a function of energetic demands [3], with body mass the single strongest predictor (larger animals often have larger home ranges), accounting for 70% of the variation among species [64]. Among similar-sized species, trophic level is a strong determinant with herbivores generally having smaller home ranges than same-size omnivores and same-size carnivores having the largest home ranges [65]. However, the current study contradicts this broad pattern, finding *P*. *volans*, with a mean body mass of 1360 g (se 0.16), to have significantly smaller home ranges than their markedly smaller sister species *P*. *minor*, with a mean body mass of 680 g (se 0.06) [9]. Therefore, in this case, differences in home range size are unlikely to reflect differences in body mass, or differences in trophic level as both *P*. *volans* and *P*. *minor* share the same folivorous diet. Therefore, we must consider other determinants.

Within a specific trophic group (both *P*. *minor* and *P*. *volans* are folivores), variations in home range size have also been attributed to the availability and distribution of resources with larger home ranges predicted where resources are less abundant or unevenly distributed [4, 66]. Variation in home range size has been described for the same taxon of gliding marsupial before [1, 67, 68] so it is established that this could be expected and related to habitat induced resource availability. Our results suggest that differences in the abundance and size of eucalyptus trees, as represented by the measure of total biomass, influences the home range sizes between these closely related greater glider species. Total biomass increases with greater tree density, diameter, and height. Large diameter trees not only increase forage opportunities but are known to have a higher probability of bearing hollows [22, 69, 70] required by greater gliders for daytime shelter.

Greater gliders have been observed to have 2–18 dens within home ranges of 0.8–4 hectares [14, 17, 71]. These dens provide not only daytime shelter but also nighttime shelter for dependent offspring, as females appear unable to glide with their young on their backs [12]. Shelter resources, including tree hollows, have been found to be influential in determining home range size in other studies [72, 73]. Smith *et al*. [17] found greater glider home range sizes 2.5–5 times larger in a low quality, mixed eucalypt-cypress forest at Barakula, than greater glider home range sizes estimated from similar studies conducted in higher quality habitats. They suggested that a lack of available den trees, with a density as low as 0.8 trees per ha, was a limiting factor in population density, consequently increasing home range size. Additionally, Eyre [74] found the number of live hollow bearing trees the strongest predictor of the abundance of greater gliders.

*Petauroides* are the smallest of the marsupial obligate folivores. The combination of a small gut capacity and the slow fermentation rates of eucalypt forage, places limitations on the dietary intake of greater gliders [75]. In moderate to high quality habitat with an abundance of preferred trees for feeding, the availability of leaves may not be the strongest driver of home range size in greater gliders. However, it is unlikely that each resource affects home range size in isolation, so the spatial distribution of resources for food and shelter in combination may also be important. Martin and Martin [73] found that mountain brushtail possums (*Trichosurus cunninghami*) had larger home range sizes when food and den sites were spatially segregated than when these resources were intermixed. We found that greater gliders predominantly preferred different tree species for food than those preferred for shelter, suggesting that the distribution of these tree species within the habitat may influence home range size.

### Home range comparisons between studies

Home range size estimates can vary markedly when calculated with different estimators such as, kernel density, MCP, or harmonic means that transform animal location data into home range estimates [55, 57, 59, 76, 77]. Notably, kernel estimates are highly sensitive to the choice of bandwidth or smoothing parameters (i.e., local bandwidth or adaptive v. global bandwidth or fixed, h_*ref*_, *ad hoc*, LSCV) [55, 57, 59, 78, 79], with differences affecting estimates of home range size and shape [59, 79, 80]. The previous studies of greater glider home range size have used differing methodology making the results of cross-study comparisons between populations unreliable (S3 Table). With extensive differences in methodology and a lack of detailed information of the methods used in some studies, cross-study comparisons of home range size should be considered cautiously [59, 81–83].

Our results for the more commonly studied *P*. *volans* were consistent with some but not all measures of home range size for this species from other published studies conducted between 1984 and 2007 (Table 4). Our mean kernel estimate (95%) of 2.0 ha falls between the estimate by Pope *et al*. [15] who recorded kernel (95%) home range estimates of 2.6 ha for males and 2.0 ha for females and Kavanagh & Wheeler [28] who recorded kernel (95%) estimate of 1.27 ha. [15]. Some inconsistencies in home range size estimates may be the result of differences in the analysis methodology used among studies (S3 Table). Kavanagh and Wheeler [28] used an adaptive kernel method with a bandwidth that varies among individual greater gliders based on location data, whereas we used a fixed kernel method with a consistent smoothing factor across all individuals at all sites. Pope *et al*. [15] used a fixed kernel method but did not report the smoothing factor or bandwidth used making direct comparisons difficult. Our home range size estimates for *P*. *volans* were considerably smaller than the 10.8 ha for males and 4.1 ha for females reported by Smith *et al*. [17]. There is some uncertainty if this population of gliders located in southern Queensland is the same taxa as *P*. *volans* [9]. The home range estimates reported by Smith *et al*. [17] are however more consistent with our results for *P*. *minor*. In addition, their study occurred in an area of low-quality habitat with a low density of greater gliders that may have resulted in larger home range sizes [17].

**Table 4:** Comparison of home range data (ha) and population density (individuals per ha) from eight studies grouped by species in descending latitude.

Study	Site Location	Kernel home range area, mean ± s.d.	MCP home range area mean ± s.d.	Population Density	Habitat description
***P*. *Volans***					
This study (2023)	Bendoc SF, north-east VIC & Wombat SF, north-west VIC	2.0 (0.42) both sexes Range 1.5 to 2.6	3.15 (1.04) both sexes Range 2.0 to 4.7	Bendoc 0.61 Wombat 2.49	Open eucalypt forests to woodlands
Henry (1984)	Boola Boola State Forest, south-eastern VIC		2.08 (0.66) polygynous males, 1.36 (0.19) monogamous males 1.25 (0.46) females 1.48 (0.59) both sexes Range 0.7–2.94	0.56	Moist sclerophyll forest
Pope *et al*. (2004)	Buccleuch State Forest, (Tumut) ACT	2.6 (0.8) males 2.0 (0.6) females		0.24–1.66	Sclerophyll remnant forest
Norton (1988)	Morton & Deua National Parks, south-east NSW		1.4 (0.2) to 2.1 (0.7) males 1.3 (0.5) to 1.5 (0.3) females	0.88–1.67	
Kavanagh & Wheeler (2004)	Coolangubra SF, south-east NSW	1.92 (0.83) males* 0.76 (0.25) females* 1.27 (0.82) both sexes*	2.03 (0.69) males* 0.81 (0.21) females* 1.35 (0.78) both sexes* Range 0.47–2.25	Not cited	Moist sclerophyll forest
Smith *et al*. (2007)	Barakula State Forest, southern QLD	10.8 (6.7) males 4.1 (2.3) females Range 1.8–17.8	11.5 (7.2) males 3.3 (2.1) females 6.8 (6.2) both sexes Range 1.4–19.3	0.1–0.36	Dry sclerophyll forest
Kehl & Borsboom (1984)	Wongi State Forest, south-eastern Queensland		2.6 (1.7) males 2.5 (1.2) females	1.2–2.3	Coastal lowland forest
***P*. *minor***					
This study (2023)	Taravale, AWC and Blackbraes NP, north-east QLD	4.79 (0.97) Range 3.7–6.5	3.15 (1.04) both sexes Range 2.0–4.7	Taravale 0.96 Blackbraes 1.92	Dry sclerophyll forest
Comport *et al*. (1996)	Taravale Station, north-east QLD	2.2 (0.1) males* 1.3 (0.1) females* Range 1.3–4.2 males, 0.9–1.7 females	1.9 (0.1) males* 0.8 (0.05) females*	3.3–3.8	Open sclerophyll forest

There has been only one other study of home range size in *P*. *minor* by Comport *et al*. [18], also at Taravale sanctuary but at a different location. They recorded a smaller home range than the current study for *P*. *minor* of 1.3 ha females, 2.2 ha males (KUD 95%) compared and consequently suggested there were no significant differences between home range sizes, of the then thought to be subspecies, *P*. *minor* and *P*. *volans*. In contrast, we found *P*. *minor* to have an estimated home range of 4.7 ha. Differences in estimates of home range size between these two studies may have resulted from dissimilarities in animal density and resource availability, in addition to probable differences in methodology. The Taravale reserve covers 59,000 ha and a variety of forest types. In his Honour’s thesis, Comport [84] describes his study site as occurring in the northern section of Taravale Station along Taravale Road. This area is predominantly wet sclerophyll forest. The current study was conducted near the southern boundary of Taravale in dry sclerophyll forest. Tree plot composition from Comport *et al*. [18] shows differences in tree communities between their study and ours. They reported *E*. *acmenoides* to be the most common tree (37%) followed by *C*. *citriodora* (28.5%). In contrast, *E*. *acmenoides* did not occur in our study transects and we rarely recorded *C*. *citriodora*. Northern forest dominated by *E*. *acmenoides* have been reported to contain the highest densities of hollows [85]. In addition, Comport *et al*. [18] reported *C*. *citriodora* as a preferred den-tree species in their survey, but this species in our study area rarely provided hollows for dens, likely because it had not reached the age (size) where hollows begin to occur. Greater glider density also differed by three-fold between the two studies with Comport *et al*. [18] reporting a density of 3.3–3.8 animals per hectare (from unpublished data) whereas we reported of 0.8–1.2 animals per hectare. Studies in mammals have shown that population density correlates negatively with home range sizes [86–88].

#### Differences in male and female home range sizes and overlap

We found no significant differences between male and female home range sizes (95% KUD) or core areas (50%) or MCP 95% within either species of *Petauroides* (Table 2). These results are consistent with some studies of *P*. *volans* [12, 14, 17], while others found males to have significantly larger home ranges in both *P*. *volans* [15, 28] and in *P*. *minor* [18]. Contradictory outcomes may result from variation in the mating system between populations across a myriad of environmental factors [12]. Henry [12] reported a mating system of facultative polygyny in greater gliders and proposed that habitat size and habitat quality produces a polygamy threshold, with polygamous groups occurring where large areas of high-quality habitat are sufficient to support more than two animals. Comport *et al*. [18] in addition to finding *P*. *minor* males to have significantly larger home range sizes than females, is the only study to find a high degree of overlap between males. These findings also contrast with our finding for *P*. *minor*.

We observed overlap of adult core areas only between males and females in both species. However, we did not collar every greater glider in our study areas and therefore, our results are not conclusive. Within-sex overlaps occurred rarely between adults and only at the outer isopleths of home range areas. This is consistent with several other greater glider studies [12, 14, 17, 28]. Notably, increases in population density can increase home range overlap between individuals [87]. The high density of the greater glider population in the Comport *et al*. [18] study may have influenced the mating system and differences between male and female home range sizes and overlap.

We recorded differences in mating season of *P*. *minor* in comparison to that documented for *P*. *volans* of births occurring from April to June [30]. We captured 12 adult female *P*. *minor* from mid-October to mid-December. These females carried joeys of varying stages of maturity from pea sized to large enough to come and go from the pouch and stages in between. Other females showed signs of lactation where offspring were left behind in dens while the females foraged. This suggests that greater gliders in this tropical habitat may mate throughout large parts of the year, although further investigation is needed.

#### Methodological considerations

This was the first study to use GPS telemetry with greater gliders. GPS telemetry allows for a larger number of accurate locations, at similar intervals and times, without the researchers’ presence, which can be limited by weather or seasonal restraints [89–92]. The use of GPS locations also eliminates the possibility of observer bias on nighttime movements. GPS precision is likely to vary among study areas; however, a single high threshold was used to screen all location data. It would have been preferable to collect GPS locations over the span of a year. Unfortunately, this was not possible due to the constraints of battery life in lightweight GPS collars. This may have resulted in an underestimated home range size if there were larger-scale seasonal shifts and preferences [93]. *Petauroides volans* have been observed to have seasonal preferences for specific species based on tree phenology; however, that study did not find seasonal differences in habitat as the number of greater gliders observed per km of transect in each habitat did not change over time [93]. This suggests that gliders may have seasonal preferences for trees producing new leaves within their existing home ranges.

### Patterns of habitat use

#### Foraging selection

Both species of greater gliders showed a clear preference for foraging in specific species of trees and in larger trees than would be expected from random selection. While 50–63% of trees at each site had a DBH of <30 cm, gliders generally avoided foraging in these smaller trees at all sites. Greater gliders at each site showed a strong preference for foraging in particular tree species, although species varied between and within species dependent on tree stand composition. *Petauroides volans* prefer eucalypt species that have relatively high concentrations of total and available nitrogen and lower concentrations of certain herbivore-deterrent plant secondary metabolites [23, 26, 94–96]. *Petauroides* preference for specific tree species, or even specific chemotypes within tree species, may outweigh their preference for particular habitat types [14, 93, 95, 97, 98]. The identification of preferred feeding trees is, therefore, critical to greater glider conservation. However, substantial differences in tree species composition and greater glider’s foraging preferences among these sites demonstrates how substantial variations can be, even between populations of the same greater glider species.

#### Shelter selection

Individual greater gliders occupied 1–6 dens, primarily in 1–2 tree species that differed at each site. Trees selected for dens varied in both species and diameter from the proportions of each available in the habitat and differed from the tree species preferred for foraging. Tree species selected for dens differed, even between populations of the same greater glider species. *Petauroides volans* at the Eastern Site (Bendoc) selected hollows in *E*. *rubida* almost exclusively for dens while those at the Western Site (Wombat) strongly preferred *E*. *viminalis*. Each of these sites lacked the tree species preferred for dens at the other site. We found *P*. *minor* at the Eastern Site (Taravale) to have 47% of dens located in *E*. *tereticornis* and 41% in *E*. *creba*. However, within the boundaries of the same reserve, but in a different forest-type, Comport *et al*. [18] found 29% of dens in *E*. *acmenoides*, followed by 25% in *C*. *citriodora* and only 14% and 13% in *E*. *tereticornis* and *E*. *crebra*, respectively. At the Western Site (Blackbraes), we found *P*. *minor* to show a preference to den in hollows in *E*. *acmenoides* (38%) but similar to Taravale, more strongly preferred *E*. *creba* (57%). While *C*. *citriodora* composed 31% of stand composition at Blackbraes, it represented only 5% of dens used by greater gliders at this site. This likely reflects a lack of maturity in the trees of this species at Blackbraes, as the mean DBH of *E*. *citriodora* recorded here was only 27.6 cm. *Eucalyptus citriodora* is known to develop hollows only at diameters >80 cm [85], so it was unlikely that hollows were present. However, the preference for this den-tree species in forests where it is more mature demonstrates the importance of retaining tree species that are likely to provide critical shelter to greater gliders in the future [71, 85].

This study found both species of *Petauroides* to have dens in hollow bearing trees with the largest diameter at each site. In keeping with findings from other studies [14, 28, 99–101] *P*. *volans* preferred den-trees with a DBH > 70 cm. No previous study has looked at den tree size preferred by *P*. *minor*. We found the DBH of den-trees selected by *P*. *minor* were considerably smaller on average than the trees selected by *P*. *volans*. However, trees with DBH > 70 cm were very rare in *P*. *minor* habitat in this study. The body mass of *P*. minor is significantly smaller than that of *P*. *volans* [9], this may help them utilize smaller hollows in trees with smaller diameters. *Eucalyptus acmenoides*, *C*. *intermedia* and *C*. *trachyphloia*, all common at our Queensland study sites, frequently develop hollows as medium sized trees between 40–80 cm in diameter [85].

In our study, we found the highest density of *P*. *minor* in survey transects dominated by *E*. *acmenoides* (Blackbraes 2.41, 3.4 animals per ha). Notably, Eyre [85] found that northern forests dominated by *E*. *acmenoides* and *Corymbia spp*. have the highest density of hollow-bearing trees, and twice that of forest dominated by *E*. *citriodora* and *E*. *creba*. Comport *et al*. [18] also reported high densities of *P*. *minor* in locations with a high abundance of *E*. *acmenoides*. As trees of smaller diameter than what are typically considered necessary for *P*. *volans* may be providing critical shelter for *P*. *minor*, they should be given consideration in future conservation and management planning for that species.

## Conclusion and management implications

Our results indicate that populations of *P*. *minor* in north Queensland have significantly larger home ranges than populations of *P*. *volans* in Victoria, although they are less than half the body mass of the larger southern species. This result is contrary to what would be predicted based on energetic requirements. Their larger home range size is likely driven by differences between northern (tropical) and southern (temperate) eucalypt-dominated habitats where tree size, biomass and hollow availability differ substantially. Understanding the use of space within and between populations can provide important information regarding factors that limit a species. This in turn can be essential for estimating population abundances and carrying capacities across their distribution, and in directing conservation and management planning. This study suggests that differences in resource availability influences home range size. Populations in north Queensland, living in areas of lower biomass may require larger home ranges to obtain sufficient resources, particularly for shelter.

This study confirms that greater gliders have strong preferences for specific tree species for browse and dens and that these preferences vary, even between geographically close populations, based on tree stand composition. This highlights the importance of understanding local habitat requirements to shape the focus of conservation efforts and habitat retention at the population scale. Additionally, the greater glider populations we studied preferred trees of the largest available diameter; however, trees with diameters >80 cm. were rare in *P*. *minor* habitats. In north Queensland, some medium-sized trees, such as *E*. *acmenoides*, are likely to be very important for the conservation of *P*. *minor*. Therefore, local estimates of home range size and habitat use must influence conservation and management plans for *P*. *minor*. The importance of large, mature trees to greater gliders’ survival is unequivocal. The relationship between tree diameter and hollow ontogeny is well documented [21, 22, 69, 71, 102, 103] with as many as 50% of trees with a diameter >80 to contain hollows [104]. Large mature trees with hollows are disappearing from many ecosystems globally [105]. The loss of these large trees is predicted to have negative cascading effects on cavity-dependent species, including greater gliders [39, 106, 107]. Conservation efforts should include retention of large hollow-bearing trees, as well as the retention of those species preferred for dens even if they have yet to reach a size for hollow formation to occur. Many Australian eucalypt trees do not develop hollows until 150–360 years after germination [21, 22, 71]. If a new cohort of trees is not available to replace those lost by dieback, clearing and wildfires, then a future lag in new hollow formation of more than 150 years could have dire consequences for the survival of greater gliders [108, 109].

## Supporting information

S1 Table Climate, geology, and vegetation information from the four sites used to estimate greater glider home range sizes and habitat use. (TIF)

S2 Table Model selection using AIC to assess the influence of environmental variables on home range size. (TIF)

S3 Table Comparison of methods used in previous publications for the estimation of greater glider home ranges. (TIF)

S1 Fig Influence of total number of locations on estimates of home range sizes. Shown here is a subset of four greater gliders from each species (two from each site). Animal locations and IDs: *P*. *minor* Eastern Site (Taravale): TG4, TG11; *P*. *minor* Western Site (Blackbraes): BG3, BG8; *P*. *volans* Eastern Site (Bendoc): EG4, EG5; *P*. *volans* Western Site (Wombat): WG1. WG3.
(TIF)
