# Supplementary figures and images for: Comparison of home range size, habitat use and the influence of resource variations between two species of greater gliders (Petauroides minor and Petauroides volans)
Source: PLoS One. 2023 Oct 19;18(10):e0286813. doi: 10.1371/journal.pone.0286813 (PMC10586627; doi:10.1371/journal.pone.0286813)

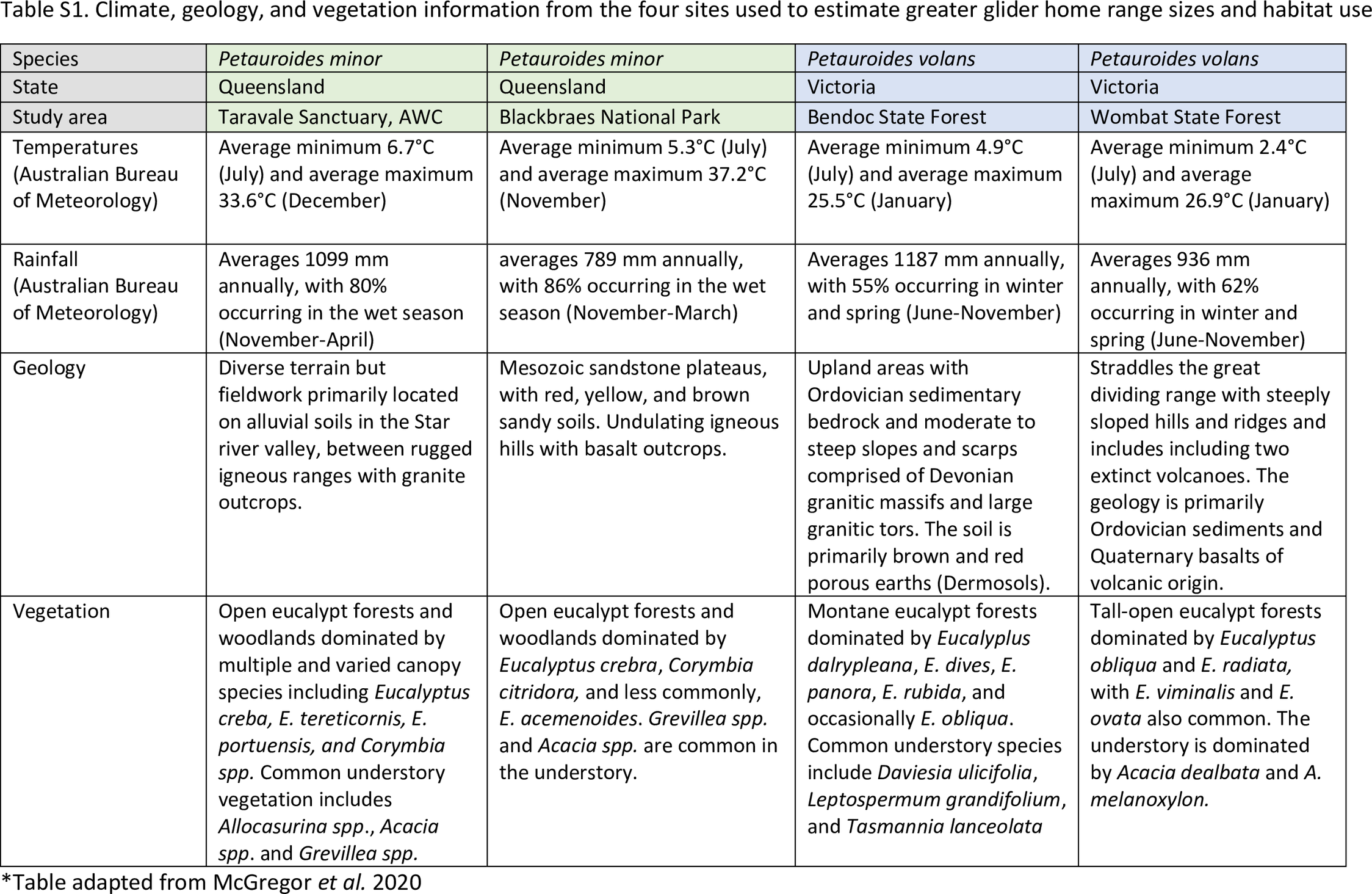

Supplement: S1 Table — (TIF) [file pone.0286813.s001.tif]

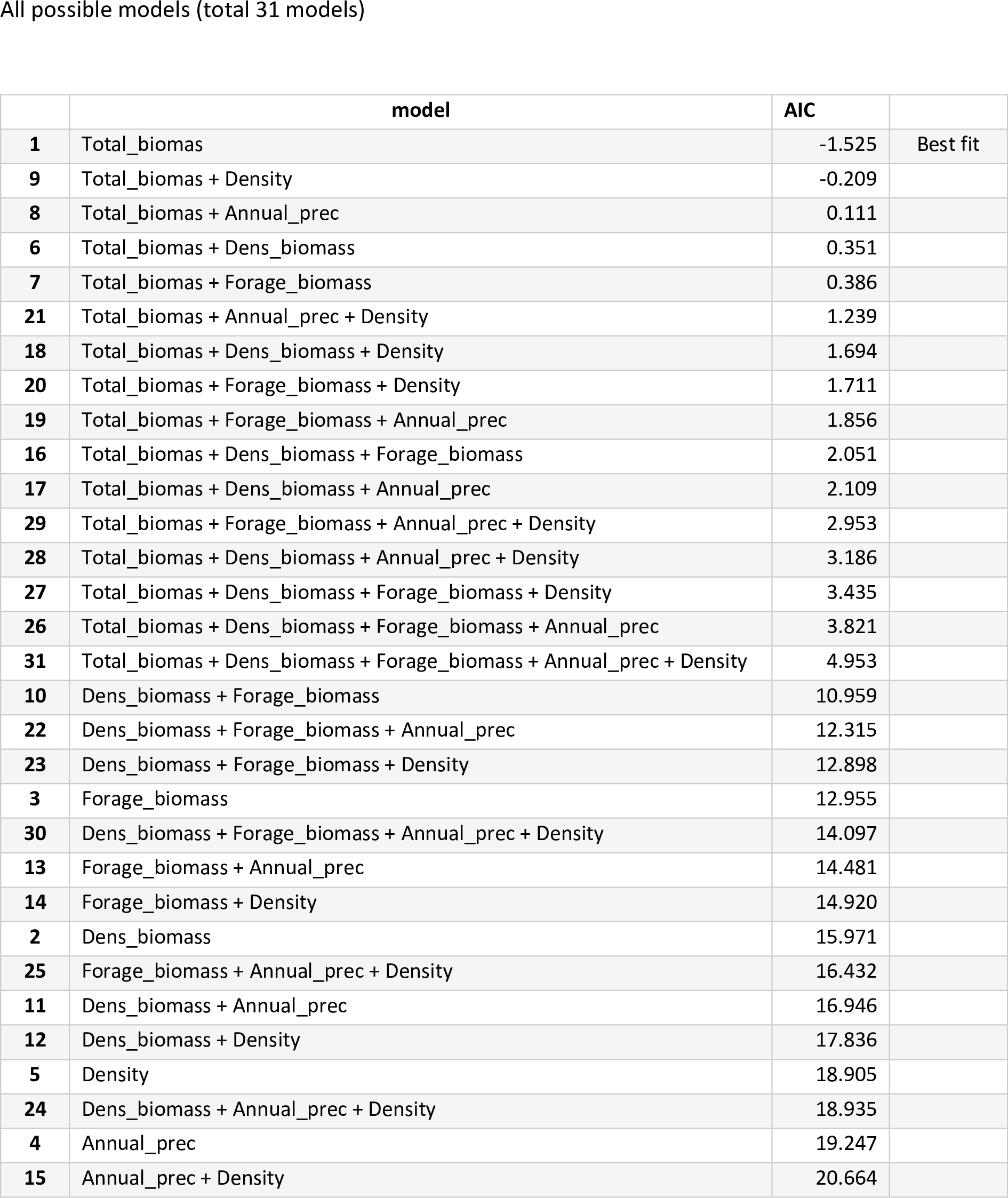

Supplement: S2 Table — (TIF) [file pone.0286813.s002.tif]

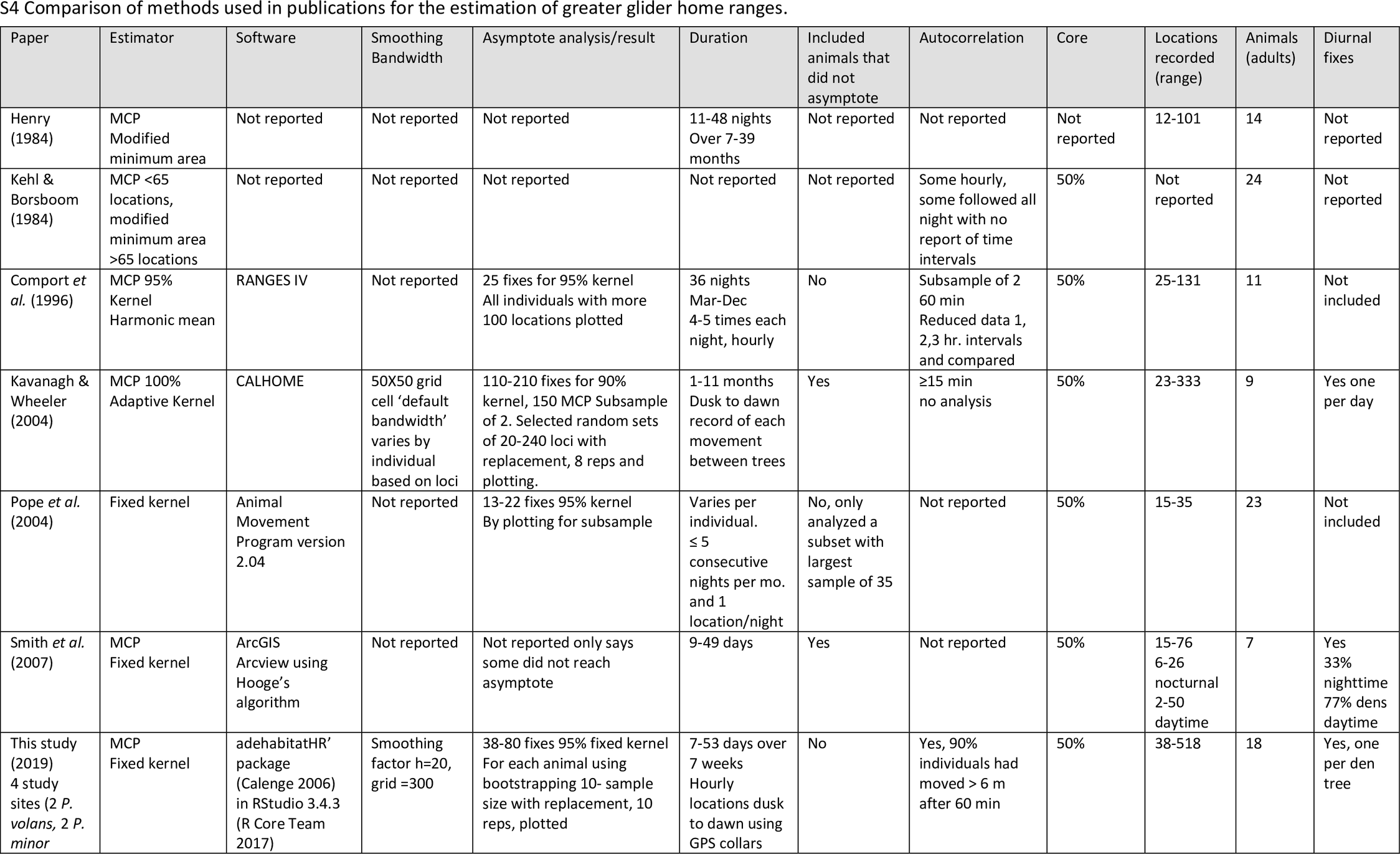

Supplement: S3 Table — (TIF) [file pone.0286813.s003.tif]

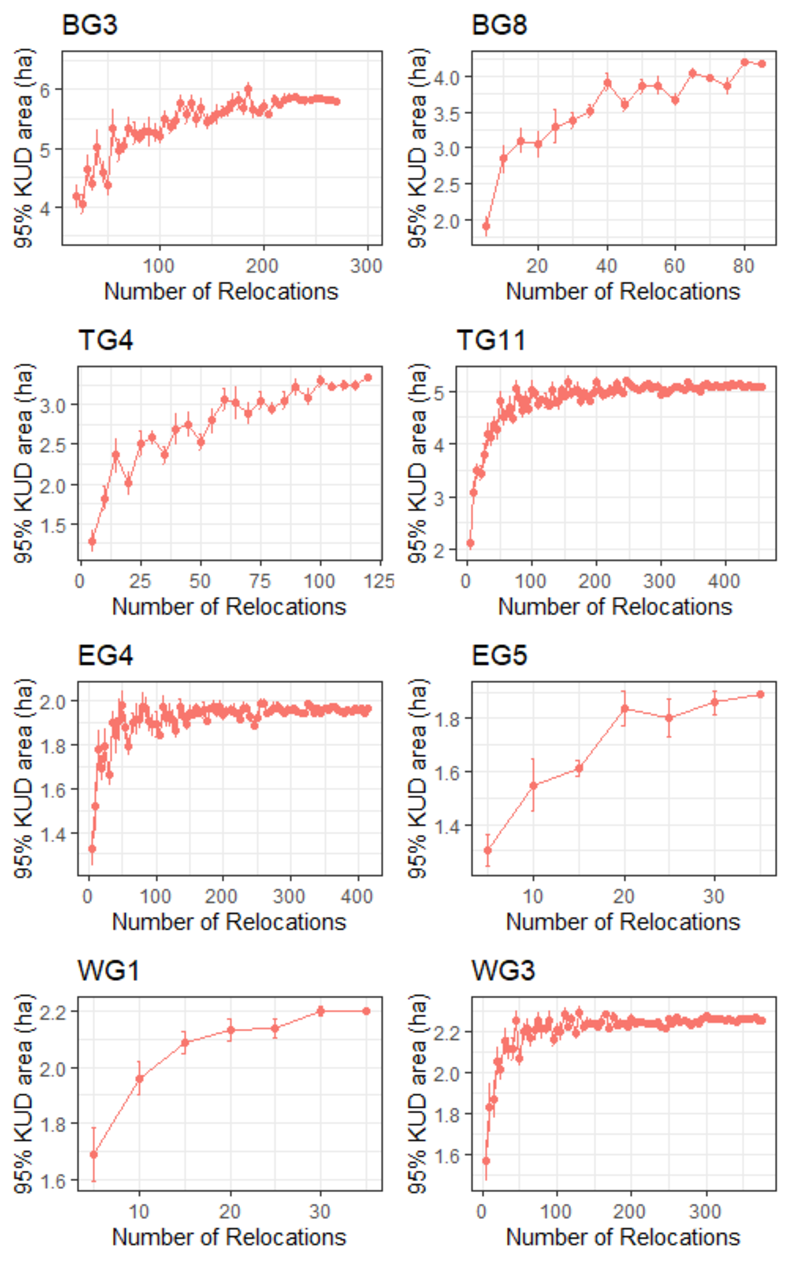

Supplement: S1 Fig — Shown here is a subset of four greater gliders from each species (two from each site). Animal locations and IDs: P. minor Eastern Site (Taravale): TG4, TG11; P. minor Western Site (Blackbraes): BG3, BG8; P. volans Eastern Site (Bendoc): EG4, EG5; P. volans Western Site (Wombat): WG1. WG3. (TIF) [file pone.0286813.s004.tif]
